# Health care providers’ perceptions regarding antimicrobial stewardship programs (AMS) implementation—facilitators and challenges: a cross-sectional study in the Eastern province of Saudi Arabia

**DOI:** 10.1186/s12941-019-0325-x

**Published:** 2019-09-24

**Authors:** Mohamed A. Baraka, Hassan Alsultan, Taha Alsalman, Hussain Alaithan, Md. Ashraful Islam, Abdulsalam A. Alasseri

**Affiliations:** 1 Pharmacy Practice Department, College of Clinical Pharmacy, Imam Abdulrahman Bin Faisal University (University of Dammam), Dammam, Saudi Arabia; 2 Pharmacy Services Department, King Fahd Hospital of the University (KFHU), Imam Abdulrahman Bin Faisal University (University of Dammam), Dammam, Saudi Arabia

**Keywords:** Antimicrobial resistance, Infections, Antibiotics, Antimicrobial stewardship programs

## Abstract

**Background:**

Infections result from invasions of an organism into body tissues leading to diseases and complications that might eventually lead to death. Inappropriate use of antimicrobials has led to development of antimicrobial resistance (AMR) which has been associated with increased mortality, morbidity and health costs. Antimicrobial stewardship (AMS) programs are designed to ensure appropriate selections of an effective antimicrobial drugs and optimizing antibiotic use to minimize antibiotic resistance by implementing certain policies, strategies and guidelines. The aim of this study was to investigate practitioners’ perceptions regarding AMS implementation and to identify challenges and facilitators of these programs execution.

**Methods:**

Cross-sectional study among health care providers in Eastern province of Saudi Arabia Hospitals. The data was collected using a survey including questions about demographic data and information about clinicians’ (physicians, pharmacists and nurses) previous experience with AMS and prescribing of antibiotics, the level of knowledge and attitudes regarding AMS programs’ implementation.

**Results:**

More than 50% of clinicians (N = 184) reported lack of awareness of AMS programs and their components, whereas 71.2% do not have previous AMS experience. The majority of clinicians (72.3%) noticed increasing number of AMR infections over the past 5 years and (69.6%) were involved in care of patients with an antibiotic-resistant infection. Around 77.2% of respondents reported that formulary management can be helpful for AMS practice and majority of respondents (79.9%) reported that the availability of pathogens and antimicrobial susceptibility testing can be helpful for AMS. Major barriers to AMS implementation identified were lack of internal policy/guidelines and specialized AMS information resources. Lack of administrative awareness about AMS programs; lack of personnel, time limitation, limited training opportunities, lack of confidence, financial issue or limited funding and lack of specialized AMS information resources were also reported 65.8%, 62.5%, 60.9%, 73.9%, 50%, 54.3 and 74.5%, respectively.

**Conclusion:**

Our study identified comprehensive education and training needs for health care providers about AMS programs. Furthermore, it appears that internal policy and guidelines need revision to ensure that the health care providers work consistently with AMS. Future research must focus on the benefit of implementing AMS as many hospitals are not implementing AMS as revealed by the clinicians. We recommend policy makers and concerned health authorities to consider the study findings into account to optimize AMS implementation.

## Background

Infections result from invasions of an organism into body tissues leading to diseases and complications that might eventually lead to death [1]. Antimicrobials have the uniqueness of treating infections, as they have the capability of not only restoring the patient’s quality of life but have also proven to be lifesaving in several severe infective conditions [1]. Inappropriate use of antimicrobials has led to development of antimicrobial resistance (AMR) which has been associated with increased morbidity, mortality and health costs [2]. Several factors have been reported to be associated with inappropriate prescribing of antimicrobials such as physicians with little experience and knowledge, uncertain diagnosis and patient influence on physician’s decision [3].

Proper use of antimicrobials leads to enhancing patient health outcomes, reducing drug consumptions and limiting the development of AMR [4]. A recent study conducted in Saudi Arabia from 2010 to 2015 revealed that most commonly used medications were antibiotics [5]. It has been reported in another study that certain classes of drugs like chemotherapeutic agents and antipsychotics are exclusively prescribed by concerned specialists, while in contrast, antibiotics are enthusiastically prescribed by all clinicians, as well as the allied healthcare personnel irrespective of their knowledge or training concerning antibiotics prescription [6]. In an attempt from the World Health Organization (WHO) to reduce AMR, several initiatives and programs have been established to educate providers about appropriate antimicrobial prescribing for appropriate indications [4]. In Saudi Arabia, the national AMS program has been activated in 2014 as a part of the pharmacy strategic plan of Ministry of Health [7]. The program started in governmental hospitals and expanded to include private hospitals 2 years later [8]. One of the challenges against AMS implementation was that majority of pharmacists were dispensing antibiotics without prescription as evidenced by national studies [9, 10]. However, this is no longer allowed after enforcing the rules that prohibit by Saudi ministry of health in 2018. It has been documented that implementation of AMS programs in the Gulf region including Saudi Arabia has led to decline in healthcare associated infections and reduction in the hospitalization period and mortality indices, in addition to tangible reduction in antibiotics cost [11]. AMS programs are designed to ensure appropriate selection of an effective antimicrobial prescribing and optimizing antibiotic use to minimize antibiotic resistance by implementing certain policies, strategies and guidelines. For the AMS program to be effective, it requires a team leader with full and ongoing financial support from the hospital administration. This also includes a staff of infectious diseases trained clinical pharmacists, as a vital component of AMS. Ongoing antibiotic education tailored to each clinical service is essential for the acceptance of AMS interventions [12]. In fact, effective hospital AMS programs need multidisciplinary engagement.

Several national and international studies revealed that inappropriate use of antimicrobials result in spreading of AMR [13–18]. Researchers have concluded that implementing AMS programs is very important to reduce antimicrobial resistance. AMS programs have been implemented in order to limit use of antibiotics, sharing of best practices and support the development of regional guidelines, also reducing the cost [13, 14]. Barriers of AMS programs include lack of collaboration and communication among healthcare providers, lack of compliance with guidelines, delay in updating knowledge, in addition to electronic system errors [2]. According to literature, general physicians are the most common prescribers of antibiotics in Saudi Arabia compared to specialists and residents [15, 16]. It was also reported that antimicrobials prescription was influenced by several factors such as; the physician’s experience especially with general physicians, patients or parents’ demand and cost. Many doctors favor treating the infections rather than colonization without being more critical in diagnosis that might lead to AMR [15, 16]. However, from the above literature survey, we highlight the presence of a gap in knowledge regarding AMS programs implementation in our country. According to the national AMS strategic plan, last stage and full implementation of AMS should have been accomplished in 2018 [8]. This constitutes a golden opportunity to conduct such kind of studies in early stages of AMS implementation. Therefore, the aims of our study were to investigate health care providers’ perceptions regarding AMS program implementation and to identify facilitators and challenges against their implementation.

## Materials and methods

### Data collection procedure

Data collection was done using self-administered questionnaires that were distributed among healthcare providers (physicians, pharmacists and nurses) in 6 large hospitals (4 governmental and 2 private more than 500 beds hospitals) in Eastern province of Saudi Arabia. To enhance data collection process, we also used online surveys (QuestionPro) that have been distributed through hospitals’ administration, pharmacy directors and nursing supervisors using emails and relevant social media pages. Several visits have been paid by the study investigators to the hospitals in addition to sending reminder emails and telephone calls to facilitate the process.

### Sample size

To determine sample size and to prove the sample size adequacy, we used power study which is a very useful and frequently used tool in medical research. Since our population size is unknown, to obtain an appropriate sample size from this population, we used the following formula [19]:

$$n = \frac{{\left( {Z_{1 - \beta } } \right)^{2} \left[ {p(1 - p)} \right]}}{{d^{2} }}$$

where n = required sample size, Z_1−β_ = Z value at power 1 − β (at power 90% this value is 1.24), p = preferred population proportion (0.5), d = margin of error (ideal value is 0.05).

Considering 90% power of test, 5% marginal error and 0.5 population proportion, use of this formula resulted in a sample size of 153.76.

In practice we may need to enroll more participants to compensate for potential missing sample or level error [20]. The formula of adjustment sample size is:

$$n_{1} = n/\left( {1 - e} \right)$$

where n = required sample size as per formula, n_1_ = is adjusted sample size, e = potential missing sample or level error.

Considering 10% dropout rate, the adjusted sample size is 170.84. This is the minimum sample size we calculated, finally, our targeted sample size for this study would be 190.

### Data collection instrument

To develop the questionnaire an in-depth literature review was carried out to assess clinicians’ perceptions about AMS programs. Among few sets of adapted questionnaires, an initial version of a structured questionnaire was drafted while receiving help of expert advisory group (other three pharmacy staff including a statistician in addition to the director of clinical pharmacy services). After compiling and adopting relevant questions from previously validated questionnaires in published literature [21–23]. To ensure the questionnaire that fulfils the study objectives and the requirements of the quality assessment domain, as well as to gain insights into its appropriateness, expert’s comments and suggestions were addressed accordingly. Whole questionnaire was divided into five parts; firstly, part A including respondents’ characteristics and experience with AMS (Cronbach’s Alpha 0.70). Second part was (named part B) covered clinicians’ perceptions (Cronbach’s Alpha 0.66 and attitudes (Cronbach’s Alpha 0.71) toward AMS programs including 24 items of five point Likert scale (responses: strongly agree, agree, uncertain, disagree and strongly disagree). To describe the factors related to clinicians antimicrobial prescribing/dispensing practices, a total of six factors with different options (allowed to choose multiple options) were undertaken and named it as part C (Cronbach’s Alpha for different factors obtained 0.61, 0.65, 0.74, 0.54, 0,84, and 0.65 for factor-1 to factor-6 respectively). Fourth part of the questionnaire was related to helpful practices that are considered as facilitators of AMS (12 items, named part D) including three responses; helpful, somewhat helpful and not helpful (Cronbach’s Alpha 0.81). Fifth and final part (named part E) of the questionnaire was to consider the major barriers of AMS implementation (eight items with five point Likert scale, Cronbach’s Alpha 0.87). To ensure face validity, the questionnaire was piloted to address all the correction about wording and ease of use, in addition to assessing the feasibility of the questionnaire. Beside this, content validity was ensured by the expert panel including three pharmacy staff, one clinicians and a statistician with the full agreement of all experts. However, data gathered from the pilot study were not included in final analysis. In terms of reliability, a number of considerations were made when designing this study to reduce threats to the reliability, including: the data collection process that was clearly documented and research procedures that were followed as per the data collection protocol during the research conduction. To ease the capture of responses from participants, the data were gathered on one occasion, and to help reduce any unintentional bias in interpreting responses from closed-ended questions were deliberately chosen for this survey design.

### Variables management

Demographic data were coded and recoded as needed. Clinicians practice (involvement, implementation, and helpful factors) with AMS were divided into three parts present, past and future. Scores were calculated (for present and past: correct answer yes = 1 and wrong answer no and don’t know = 0; as well as for future: correct answer helpful = 1 and wrong answer somewhat and not helpful = 0) for measuring clinicians practice and divided into two categories; good practice and bad practice using k-mean cluster statistical method. To calculate clinicians’ attitude and perception score five point Likert scale was used as 5 to 1 for strongly agree, agree, uncertain, disagree and strongly disagree respectively. Finally using k-mean cluster statistical method, it was divided into two categories; good perception for higher score and bad perception for lower score. Major barriers’ variables were divided into three categories; agree (strongly agree and agree), uncertain (as usual) and disagree (disagree and strongly disagree).

### Statistical analysis

Statistical analyses were carried out using Statistical Package for Social Sciences (IBM SPSS version 23.0) and Excel software. Data ware checked and cleaned using informal technique. As this was online survey (QuestionPro) missing cases were found, some of them were treated using ‘*last*-*observation*-*carried*-*forward method’* (which is commonly used in pharmaceutical research) and some of them were excluded them from final analysis. Descriptive statistics was used to describe the study variables and items and frequencies and corresponding percentages have been reported. For statistical significance p < 0.05 was considered as acceptable range of type-I error.

## Results

Data were analyzed for a total of 184 clinicians where 99 participants, more than half of them (53.8%) were pharmacists, 35.8% were physicians, and 10.9% were nurses. Overall, 56% were males whereas 44% were females. Same proportion found for the respondents whose age was less than 30 years. Regarding years of practice, 37% of the clinicians had less than 3 years of clinical experience. Only 16% of respondents reported that their experience exceeds 10 years. When we studied average of continuous medical education (CME) hours/year results, it revealed that more than 70% of the respondents (71%) had more than 20 continuous medical education hours per year (Table 1).

**Table 1:** Descriptive statistics for socio-demographic characteristics of respondents (n = 184)

Characteristics	Group	Frequency (n)	Percentage (%)
Gender	Male	103	56.0
	Female	81	44.0
Age (year)	Less than 30	103	56.0
	31–40	56	30.4
	41 and above	25	13.6
Nationality	Saudi	154	83.7
	Non-Saudi	30	16.3
Region	Eastern of Saudi Arabia	178	96.7
	Others	6	3.3
Profession	Nurses	20	10.9
	Physicians	65	35.3
	Pharmacists	99	53.8
Country of last professional degree	Saudi Arabia	149	81.0
	Others	35	19.0
Working hours (per day)	Less than 8 h	50	27.2
	8 h and more	134	72.8
Years of practice	Less than 3 years	68	37.0
	3–6 years	50	27.2
	6–10 years	36	19.6
	More than 10 years	30	16.3
Average of CME (continuous medical education) hours/year	Less than 20	53	28.8
	20 and above	131	71.2

Table 2 represents the results of previous involvement and experience with antimicrobial resistance and antimicrobial stewardship (AMS) programs. Results demonstrated that among all, more than 50%, 52.7% clinicians reported they are not aware of antimicrobial stewardship (AMS) programs and their components, whereas 71.2% do not have previous AMS experience. But many clinicians (72.3%) noticed increasing number of antimicrobial-resistant infections over last 5 years and (69.6%) were involved in care of patients with an antibiotic-resistant infection. Almost 66% (65.8%) of healthcare providers agreed that their hospitals provide guidelines/policy for diagnosis and management of patients with infective problems, and nearly same percentage (69.6%) of respondents ensure that they follow the recommendations of their hospital about antimicrobial guidelines/policy, while only a very low number of clinicians (10.9%) received a specialized training in AMS programs.

**Table 2:** Previous involvement and experience with antimicrobial resistance and antimicrobial stewardship (AMS) programs

Items	Responses	
	Yes (n & %)	No (n & %)
Are you aware of antimicrobial stewardship (AMS) programs and their components?	87 (47.3)	97 (52.7)
Do you have previous AMS experience?	53 (28.8)	131 (71.2)
Have you noticed increasing number of antimicrobial-resistant infections over last 5 years?	133 (72.3)	51 (27.7)
Have you ever been involved in care of patients with an antibiotic-resistant infection?	128 (69.6)	56 (30.4)
Have you worked in health care facilities with AMS programs?	51 (27.7)	133 (72.3)
Have you received specialized training in AMS programs?	20 (10.9)	164 (89.1)
Does your hospital provide guidelines/policy for diagnosis and management of patient with infective problems?	121 (65.8)	63 (34.2)
Do you follow the recommendations of your hospital antimicrobial guidelines/policy?	128 (69.6)	56 (30.4)
Do you believe that antimicrobials are used too much in clinical settings?	146 (79.3)	38 (20.7)
Have you ever been forced to choose antibiotics you feel are inappropriate because of the antibiotic approval program?	65 (35.3)	119 (64.7)
Is the infectious diseases service in your hospital easily accessible and helpful?	114 (62.0)	70 (38.0)
Does your hospital contain drug information services/centers?	113 (61.4)	71 (38.6)

The results of Presence and implementation of specific antimicrobial stewardship (AMS) program policies were presented in Table 3. Most of the respondents (70.1%) reported that the policy requiring prescribers to document indication for antibiotic. Almost three-fourth (73.4%) of the respondents agreed that individual patient care is improved by having an antibiotic approval program. Almost 70% (70.7%) of the respondents found having to call for approval makes the team think more carefully about choosing an antibiotic. However, 66.8% of the respondents found that is not the primary purpose of the antibiotic approval program is to reduce the amount of money the hospital spends on antibiotics.

**Table 3:** Presence and implementation of specific antimicrobial stewardship (AMS) program policies

Items	Responses	
	Yes (n & %)	No (n & %)
Policy requiring prescribers to document indication for antibiotic	129 (70.1)	55 (29.9)
Individual patient care is improved by having an antibiotic approval program	135 (73.4)	49 (26.6)
Having to call for approval makes the team think more carefully about choosing an antibiotic	130 (70.7)	54 (29.3)
The primary purpose of the antibiotic approval program is to reduce the amount of money the hospital spends on antibiotics	61 (33.2)	123 (66.8)
The clinician who is seeing the patient is in a more appropriate position to pick the correct antibiotic than someone on the phone who has never seen the patient	126 (68.5)	58 (31.5)

Opinion on different practice which can be helpful as facilitators of AMS were obtained and expressed in Table 4. Results demonstrated that 77.2% of respondents have agreed that formulary management can be helpful for AMS practice. a large percentage of respondents reported that the availability of pathogens and antimicrobial susceptibility testing can be helpful for AMS practice and the stated results were (79.9%). Real-time feedback, IT department support, time and Incentives/funding were found almost similar percentage 58.7%, 53.3% and 54.3%, respectively to support helpful opinion on AMS practice. Finally, almost 70% (69.6%) of clinicians reported that didactic education is helpful for practices in AMS.

**Table 4:** How helpful the following practices are as facilitators of AMS

Items	Responses (n & %)		
	Helpful	Somewhat helpful	Not helpful
Formulary management (i.e. selection of antimicrobials for inclusion on hospital formulary based on efficacy, toxicity and cost) is essential	142 (77.2)	40 (21.7)	2 (1.1)
Real-time feedback (contact from a pharmacist by page/phone regarding an antimicrobial prescription) should be provided	108 (58.7)	60 (32.6)	16 (8.6)
Didactic education (lectures from infectious disease specialists and pharmacists) and training should be available	128 (69.6)	48 (26.1)	8 (4.3)
Supplemental online AMS resources Clinical guidelines should be accessible	136 (73.9)	43 (23.4)	5 (2.7)
Annual antibiogram (available electronically while prescribing/dispensing) should be prepared and circulated to prescribers/dispensers	137 (74.5)	42 (22.8)	5 (2.7)
Availability of AMS team	134 (72.8)	46 (25.0)	4 (2.2)
Leadership support	116 (63.0)	63 (34.2)	5 (2.7)
IT department support	98 (53.3)	69 (37.5)	17 (9.2)
Time and incentives/funding	100 (54.3)	66 (35.9)	18 (9.8)
Addition of antibiotic indication field (which lists numerous indications and includes an option for other) to the computerized prescription/order entry	128 (69.6)	51 (27.7)	5 (2.7)
Pharmacists suggestion for an alternative therapeutic agent for treatment of infection	121 (65.8)	55 (29.9)	8 (4.3)
Availability of pathogens and antimicrobial susceptibility test results	147 (79.9)	35 (19.0)	2 (1.1)

Almost two-third (74.5%) of respondent agreed that the major barriers could be the lack of internal policy/guidelines and lack of specialized AMS information resources. Response on other barriers like: administrative awareness about AMS program; lack of personnel, time limitation, limited training opportunities, lack of confidence, financial issue or limited funding and lack of specialized AMS information resources were found 65.8%, 62.5%, 60.9%, 73.9%, 50%, 54.3 and 74.5%, respectively. It was noticed that maximum supporting barrier on AMS reported (74.5%) on two items firstly, lack of internal policy/guidelines and secondly, lack of specialized AMS information resources (Table 5).

**Table 5:** Major barriers of AMS

Items	Responses (n & %)		
	Agree	Natural	Disagree
Lack of internal policy/guidelines	137 (74.5)	36 (19.6)	11 (6.0)
Administration not aware of AMS program	121 (65.8)	43 (23.4)	20 (10.9)
Lack of personnel	115 (62.5)	50 (27.2)	19 (10.3)
Limited time	112 (60.9)	48 (26.1)	24 (13.0)
Limited training opportunities	136 (73.9)	35 (19.0)	13 (7.1)
Lack of confidence	92 (50.0)	62 (33.7)	30 (16.3)
Financial issue or limited funding	100 (54.3)	48 (26.1)	36 (19.6)
Lack of specialized AMS information resources	137 (74.5)	38 (20.7)	9 (4.9)

For assessment to the perceptions toward antimicrobial stewardship programs, 19 questions were utilized and represented in the Table 6. Most of them agreed that Antimicrobial resistance is a problem worldwide (95.1%), whereas (83.7%) agreed that poor infection control practices by healthcare professionals causes antimicrobial resistance. A great proportion of respondents (78.8%) were supporting the statement that AMS programs reduce problems of antimicrobial resistance. The majority of respondents agreed on the importance of generating a policy to limit prescribing of antimicrobials, also they agreed that there must be a team consisting of an infectious disease specialist physician and pharmacist by the proportion 84.8%, 85.9%, respectively. In addition, almost 80% of respondents (77.2%) rejected the statement that the Health care professionals other than prescribers do not need to understand AMS.

**Table 6:** Clinicians’ perceptions toward antimicrobial stewardship programs

Items	Responses	
	Disagree (n & %)	Agree (n & %)
Antimicrobial resistance is a problem worldwide	9 (4.9)	175 (95.1)
Antimicrobial resistance is a problem in my daily practice	48 (26.1)	136 (73.9)
Poor infection control practices by healthcare professionals causes antimicrobial resistance	30 (16.3)	154 (83.7)
Prescribing broad-spectrum antibiotics when there are equally effective narrower-spectrum antibiotics increases antibiotic resistance	35 (19.0)	149 (81.0)
It is always better to over-prescribe antibiotics than under-prescribe?	138 (75.0)	46 (25.0)
Antimicrobials might develop allergy leading to death	55 (29.9)	129 (70.1)
AMS programs reduce problems of antimicrobial resistance	39 (21.2)	145 (78.8)
AMS will help reduce hospitalization	41 (22.3)	143 (77.7)
Optimization of child and adult dose is essential	18 (9.8)	166 (90.2)
If symptoms improve before the full course of antimicrobial is completed, your patient can stop taking it	147 (79.9)	37 (20.1)
Everyone should be able to buy antibiotics without a prescription	146 (79.3)	38 (20.7)
Improving antimicrobial prescribing should be an organizational priority	26 (14.1)	158 (85.9)
A policy that limits the prescribing of selected antimicrobials to certain clinical indications via an approval process should be introduced at the hospital	28 (15.2)	156 (84.8)
Locally developed guidelines for antimicrobials would be more useful to me than national guidelines	84 (45.7)	100 (54.3)
A team consisting of an infectious disease specialist physician and pharmacist providing individualized antimicrobial prescribing advice and feedback would assist with antimicrobial selection	26 (14.1)	158 (85.9)
A computer application which gives advice on selection and duration of antimicrobial therapy for specific clinical conditions would be clinically useful	38 (20.7)	146 (79.3)
AMS education is provided for all staff involved in antimicrobial ordering, dispensing, administration, and monitoring	34 (18.5)	150 (81.5)
Health care professionals other than prescribers do not need to understand AMS	142 (77.2)	42 (22.8)
Pharmacists have a responsibility to take a prominent role in AMS and infection-control programs in the health system	34 (18.5)	150 (81.5)

For assessment to the attitudes toward antimicrobial stewardship programs, 5 questions of Likert scale were used. For the first question “I am concerned about antibiotic resistance in my hospital when I prescribe or dispense antibiotics” 73.4% of clinicians agreed on this statement. Almost two-thirds of the respondents (58.7%) agreed with the second question “I feel confident about my knowledge and practice in the area of antimicrobial prescribing”. More than 80% (83.2%) agreed on “I would be willing to participate in any activities to improve the quality of antimicrobial use at my hospital”. Around 64% and 78% clinicians agreed with the fourth question “I take part in antimicrobial-awareness campaigns to promote the optimal use of antimicrobials and the fifth question” “I educate patients on the use of antimicrobials and resistance-related issues” respectively (Table 7).

**Table 7:** Clinicians’ attitudes toward antimicrobial stewardship programs

Items	Responses	
	Disagree (n & %)	Agree (n & %)
I am concerned about antibiotic resistance in my hospital when I prescribe or dispense antibiotics	49 (26.6)	135 (73.4)
I feel confident about my knowledge and practice in the area of antimicrobial prescribing	76 (41.3)	108 (58.7)
I would be willing to participate in any activities to improve the quality of antimicrobial use at my hospital	31 (16.8)	153 (83.2)
I take part in antimicrobial-awareness campaigns to promote the optimal use of antimicrobials	66 (35.9)	118 (64.1)
I educate patients on the use of antimicrobials and resistance-related issues	39 (21.2)	145 (78.8)

## Discussion

The study assessed the perception of health care providers regarding implementation, facilitators and barriers of AMS implementation in Eastern province of Saudi Arabia hospitals. It is obvious that without understanding health care providers’ perceptions and attitudes regarding AM, any educational interventions concerning AMR and AMS programs will lead to failure of these initiatives [1].

Similar to health care providers from other parts of the world, the vast majority of our respondents agreed that AMR is a global problem [17]. Perceiving AMR as a major health problem is the first step in resolving the problem. Otherwise, it will be very challenging to change the clinicians practice towards AMR prevention [21]. In addition to that, our results conform with other studies that using antimicrobials unnecessarily, using antimicrobials without physician prescription (self-medication) and not completing the full course of antimicrobials are key factors contributing to antimicrobial resistance [24, 25]. In response to this and based on clinicians’ responses here and in previously published literature, we may argue that educating healthcare providers on selecting appropriate antimicrobial therapy, providing local antimicrobial guidelines and consulting with infectious diseases experts may be warranted for successful implementation of AMS [21, 26, 27]. Educating health care providers on providing rational antimicrobial prescribing was also recommended by the WHO in order to prevent AMR [26, 27]. Our study findings revealed also that availability of pathogens and antimicrobial susceptibility testing can be helpful for AMS practice and that Real-time feedback, IT department support, time and Incentives/funding were perceived as facilitators for AMS practice.

Most of our respondents agreed that antimicrobial stewardship programs will reduce problems of antimicrobial resistance. Although there are many studies agreed that AMS is a key strategy to improve the appropriateness of antimicrobial use [2–4]. In contrast, the majority of respondents had no previous AMS experience and only 10.9% of them had received specialized training in AMS programs. This might be because implementation of AMS in Saudi Arabia is still new. The majority of our respondents agreed that policy requiring prescribers to document indication for antibiotic is a key factor for implementing (AMS) programs.

Since the majority of health care providers think that lack of internal policy/guidelines is a barrier for AMS implementation, for that reason, we think that every hospital should generate their internal policy or their internal guidelines if the international guidelines are not applicable for their practice. In addition to this and in concordance with our findings, it was also recommended by other studies to provide local surveillance to identify bacteria responsible for nosocomial infections and development of AMR [13, 14]. Although this plays a vital role in developing the local guidelines, this does not always translate into utilization for such guidelines in practice [23]. Furthermore, lack of specialized AMS information resources is a major challenge for proper AMS implementation so the hospitals should initiate committee responsible to create policy focusing on infections management [5]. In addition to this, local drug information centers should expand their role to provide specialized antimicrobial precise information to empower clinicians while making their choices regarding antimicrobials prescribing, dispensing and administration [23].

Clinicians recognize Antimicrobial resistance as a global problem and agreed that poor infection control practices by healthcare providers cause AMR. They agree that AMS programs reduce developing AMR. They are also supporting the team work in AMS implementation and believe that these teams should include an infectious disease specialist physician and pharmacist. However, they were against the statement that the Health care professionals other than prescribers do not need to understand AMS.

Many of clinicians assure that rational selection of drugs list to the hospitals formulary management is a facilitator for AMS. Most of respondents support that antimicrobial susceptibility test results, Supplemental online AMS resources Clinical guidelines should be accessible as this would be helpful for proper AMS implementation. A recent study conducted in Saudi Arabia by AlKhamees et al. was in line with our result in the importance of interprofessional networks and collaborations between healthcare providers, such as committee work and guideline composition as facilitators for AMS programs implementation [5].

The good news in our study is that clinicians have positive attitudes towards AMS implementation as they feel confident about their knowledge and practice in the area of antimicrobial prescribing. They will be happy to participate in any activities to improve the quality of antimicrobial use at their hospitals. Clinicians are also willing to take part in antimicrobial-awareness campaigns to promote the optimal use of antimicrobials and are willing to educate patients on the use of antimicrobials and resistance-related issues.

Although our study findings shed light on understanding clinicians’ perceptions and attitudes regarding successful implementation of AMS programs, while interpreting our study results, a number of limitations exist. First, the study was conducted in only six hospitals in the Eastern region in Saudi Arabia, therefore, we should be careful generalization cannot be guaranteed to the whole nation without conducting such studies on larger scale and including other regions in the kingdom. Second, the small sample size and inclusion of only two private hospitals may not reflect the real situation in all private hospitals and hence limits generalizability of the study findings. Third, it would have been more interesting to investigate differences among the different clinicians’ groups such as physicians versus pharmacists and nurses in terms of perceptions and attitudes towards AMS implementation. However, we are planning to assess these differences in addition to factors affecting clinicians’ attitudes in coming studies.

In fact, further studies are warranted to investigate the real reasons behind the lack of knowledge, experience and skills as revealed by clinicians and that are required for AMS successful conduction in our region.

## Conclusion

Our study identified a comprehensive education and training needs for health care providers about AMS programs. It appears that internal policy and guidelines need revision to ensure that the health care providers work consistently with AMS requirements. More studies are warranted to further enhance our understanding about AMS programs optimization to benefit from the positive attitude of the clinicians towards this issue. Future research must focus on predictors of clinicians’ perceptions and attitudes and the benefits of implementing AMS. The study showed that hospitals are not fully implementing AMS as revealed by the clinicians, therefore, we recommend policy makers and concerned health authorities to consider our study into account when they planning to implement AMS.

## Data Availability

All data and materials are available upon request.
